# Nodal frozen section + elective neck dissection as an alternative to sentinel lymph node biopsy for the management of cT1-2N0 oral squamous cell carcinoma patients: a viability and accuracy study

**DOI:** 10.1007/s00432-023-04941-6

**Published:** 2023-06-06

**Authors:** Salvatore Battaglia, Salvatore Crimi, Eliana Piombino, Loredana Villari, Claudia Maugeri, Giuseppe Minervini, Marco Cicciù, Alberto Bianchi

**Affiliations:** 1 Department of Biomedical and Surgical and Biomedical Sciences, Catania University, 95123 Catania, CT Italy; 2 Pathology Unit San Marco Hospital, San Marco Hospital, Catania, Italy; 3 Division of Maxillofacial Surgery Surgical Science dpt., Città Della Salute e Delle Scienze Hospital, University of Turin, Turin, Italy; 4 Multidisciplinary Department of Medical-Surgical and Odontostomatological Specialties, University of Campania “Luigi Vanvitelli”, 80121 Naples, Italy

**Keywords:** Oral cancer, Intra-operative frozen section, Sentinel lymph node biopsy (SLNB), Elective neck dissection

## Abstract

**Purpose:**

Oral Squamous Cell Carcinoma (OSCC) is characterized by a high aggressiveness and a tendency to metastasize. The management of the neck in cT1-2N0 patients c follows three strategies: watchful waiting, elective neck dissection (END) or sentinel lymph node biopsy (SLNB). The aim was to assess the viability of intraoperative frozen sections of the nodes of cT1-2N0 to spot occult metastases as an alternative to SLNB, performing a modified radical neck dissection (MRND) in intraoperatively positive patients. *Methods:* The patients were treated at the Maxillo-Facial Surgery Unit of Policlinico San Marco of Catania between 2020 and 2022. END was performed in all patients, including frozen section examination of at least one clinically suspicious node per level. In case of positivity after frozen section examination, neck dissection was extended to levels IV and V. *Results:* All frozen sections were compared with a definitive test after paraffin inclusion. During surgery, 70 END were performed, and 210 nodes were analyzed with frozen sections. Among the 70 END, 52 were negative after frozen Sects. (156 negative nodes), and surgery was ended. Five of the 52 negative ENDs resulted in pN + after paraffin inclusion (9.6%), which underwent postoperative adjuvant treatment. The sensibility of our END + frozen section method was 75%, while the specificity of our test was 94%. The negative predictive value was 90,4%.

**Conclusions:**

Elective neck dissection + intraoperative frozen section could be an alternative to SLNB to spot occult nodal metastases in cT1-2N0 OSCC due to the opportunity to perform a one-step diagnostic/therapeutic procedure.

## Introduction

 Oral Squamous Cell Carcinoma (OSCC) is the sixth most frequent tumour worldwide, and its prevalence is assessed at 3% of all tumours (Bray et al. 2011).

OSCC is the most frequent malignancy of the oral cavity. It is characterized by a high local aggressiveness and a tendency to locoregional metastases (Bray et al. 2011).

The presence of nodal metastasis is considered the most critical negative predictive factor for the prognosis of the patients, reducing the overall survival by 50% (Ren et al. 2015a; Ren et al. 2014; Ren et al. 2015b; Hamoir et al. 2014). The early detection of neck lymph node disease is essential for better survival (Fang et al. 2020; Ren et al. 2015c).

Palpation has a low sensitivity in detecting lymph node metastases and proved to be inferior compared to conventional imaging techniques, such as ultrasonography (US), computerized tomography (CT) and magnetic resonance imaging (MRI) and Fluorine-18-fluorodeoxyglucose positron emission tomography (FDG PET). Sometimes the lymph nodes are negative to clinical examination, but there are micrometastases in the lymph nodes after pathological examination after neck dissection, called occult lymphatic metastasis (Rose et al. 2011).

 Elective neck dissection (END) is recommended for clinically N0 oral cavity carcinomas. In contrast, a modified radical neck dissection (MRND) in patients with a clinically positive cervical lymph node achieves better disease-free survival with minimal post-operative co-morbidity (Ren et al. 2015a).

However, lymph node metastases are not the only cause of poor prognosis. The discrepancy between the successful initial treatment of the tumour and poor long-term forecast is also related to the patient’s comorbidities, recurrent primary tumours, second primaries, and distant metastases developing in the further course of the disease (Rose et al. 2011).

Although the importance of neck dissection in cN + patients is recognised worldwide, its role in clinically negative neck patients is still debated (D’Cruz et al. 2015; Fakih et al. 1989). Furthermore, according to the literature, around 30% of OSCC patients assessed pre-operatively as cN0 were revealed to be pN + after surgery (Guidelines 2022).

Surgery is still the preferred treatment; neck dissection is essential in treating OSCC. According to the updated NCCN guidelines, the management of the neck in cT1-2N0 patients could follow three different strategies: watchful waiting, elective neck dissection (END) or sentinel lymph node biopsy (SLNB) (Guidelines 2022).

The watchful-waiting approach can avoid an additional surgical procedure in up to 70% of patients who eventually are found to be node-negative on histopathological analysis (Fakih et al. 1989). The SLNB are the first lymph nodes that receive metastases from the primary tumour (Liu et al. 2017). The biopsy of sentinel lymph nodes is routinely used to manage breast cancer, colon cancer, and cutaneous malignant melanoma (CMM). In the last decade, sentinel lymph node biopsy (SLNB) was introduced in OSCC as a less invasive alternative with lower morbidity rates than END. In early stage OSCC, the most recent meta-analysis reported a pooled sensitivity of 87% and a negative predictive value of 94% for the SLNB procedure in detecting occult metastasis (Liu et al. 2017). Because of the low invasiveness and high accuracy rates, the SLNB procedure is implemented in many national head and neck guidelines.

Some Authors have recently demonstrated that performing an END in cT1-2N0 OSCC patients is related to a lower risk of recurrence and higher rates of disease-free survival (DFS) and overall survival (OS) compared to patients treated with watchful waiting and eventual therapeutic neck dissection. On the other hand, in experienced centres, SLNB could be a good alternative for spotting occult neck metastases (Ross et al. 2002). However, no studies in the literature could demonstrate a statistically significant difference between the two strategies in terms of DFS and OS (Sparano et al. 2004).

Moreover, SLNB is not affordable in every Hospital, since it needs experienced teams and Nuclear Medicine Department (Liu et al. 2017). The current study aimed to assess the accuracy and viability of intraoperative frozen sections of the nodes of cT1-2N0 patients to spot occult metastases as an alternative to SLNB, performing a modified radical neck dissection (MRND) in the intraoperatively positive patients and to assess the sensibility, specificity and the predictive value of this approach.

The union of the elective neck dissection with the execution of the frozen section give the possibility surgeon to remove the tumour and to modify the operating planning without lengthening the surgical time.

## Materials and methods

The patients of our study were treated at the Maxillo-Facial Surgery Unit of Policlinico San Marco, Catania, Italy, between June 2020 and June 2022.

Each patient provided written informed consent for involvement. Informed consent was obtained before data collection.

The inclusion criteria were the following:

- Age > 18 years;
- Primary OCSCC;
- cN0 at presentation.
- The exclusion criteria were:
- Age < 18 years;
- cN + patients at presentation;
- Relapse or secondary tumour at presentation.

All patients underwent preoperative imaging assessment with a CT scan and were diagnosed with incisional biopsy. Characteristics of the tumour at the incisional biopsies were assessed, such as grading and pattern of invasion when possible. Other parameters, such as DOI, were evaluated in the surgical specimen after surgery. END was performed in all patients, including frozen section examination of at least one clinically suspicious node per level. *The features of suspicious nodes analyzed were: size, shape, necrosis, and extra-capsular spread* (Ross et al. 2002). In case of positivity after frozen section examination, neck dissection was extended to levels IV and V.

### Intra-operative END Frozen section

The intra-operative procedure required two dedicated expert pathologists assisted by a laboratory technician performing frozen sections.

The first step involved macroscopic evaluation; the small nodes (< 5 mm) were frozen completely, the larger nodes were cut in two halves, and the material was frozen.

**Fig. 1:**
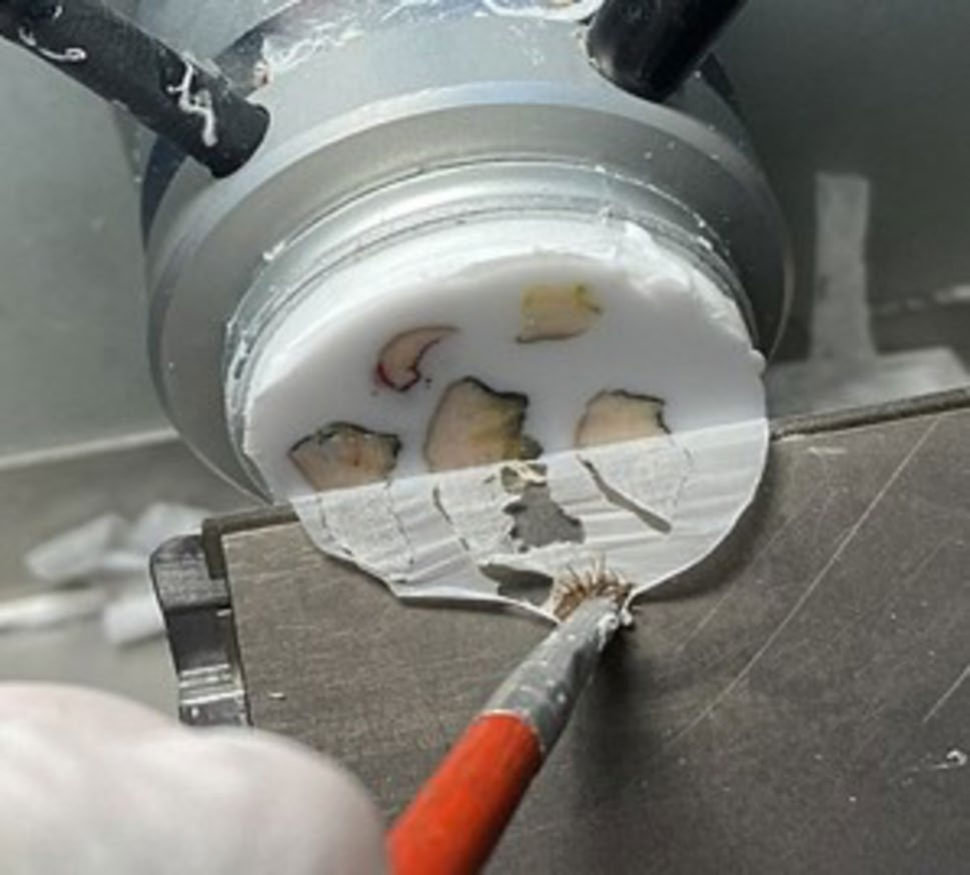
Frozen section of END nodes performed with the − 20 °C cryostat

The lab technician cut the sections at -20° C using a cryostat (Fig. 1). As a protocol, for each frozen block, four sections, stained with rapid hematoxylin and eosin, were evaluated for metastasis under the microscope. The fast hematoxylin–eosin staining procedure was performed in each case: the slide was immersed in hematoxylin for 1 min. After washing, a dip in 1% acid alcohol for differentiation, followed by “bluing” under tap water for 2 min. Subsequently, a 1% aqueous eosin was dropped, followed by mounting.

The sections were evaluated under a microscope by both dedicated pathologists to reduce the possibility of false negatives.

The intra-operative diagnosis was completed (average time 20 min), and it was immediately communicated by telephone to the surgeons and inserted into the reporting system.

All intraoperative frozen sections underwent the analysis of the paraffine embedded section.

## Results

Seventy patients met the inclusion criteria (M:F = 44:26) with an average age of 67,5 years old (range 18–91 yy).

**Fig. 2:**
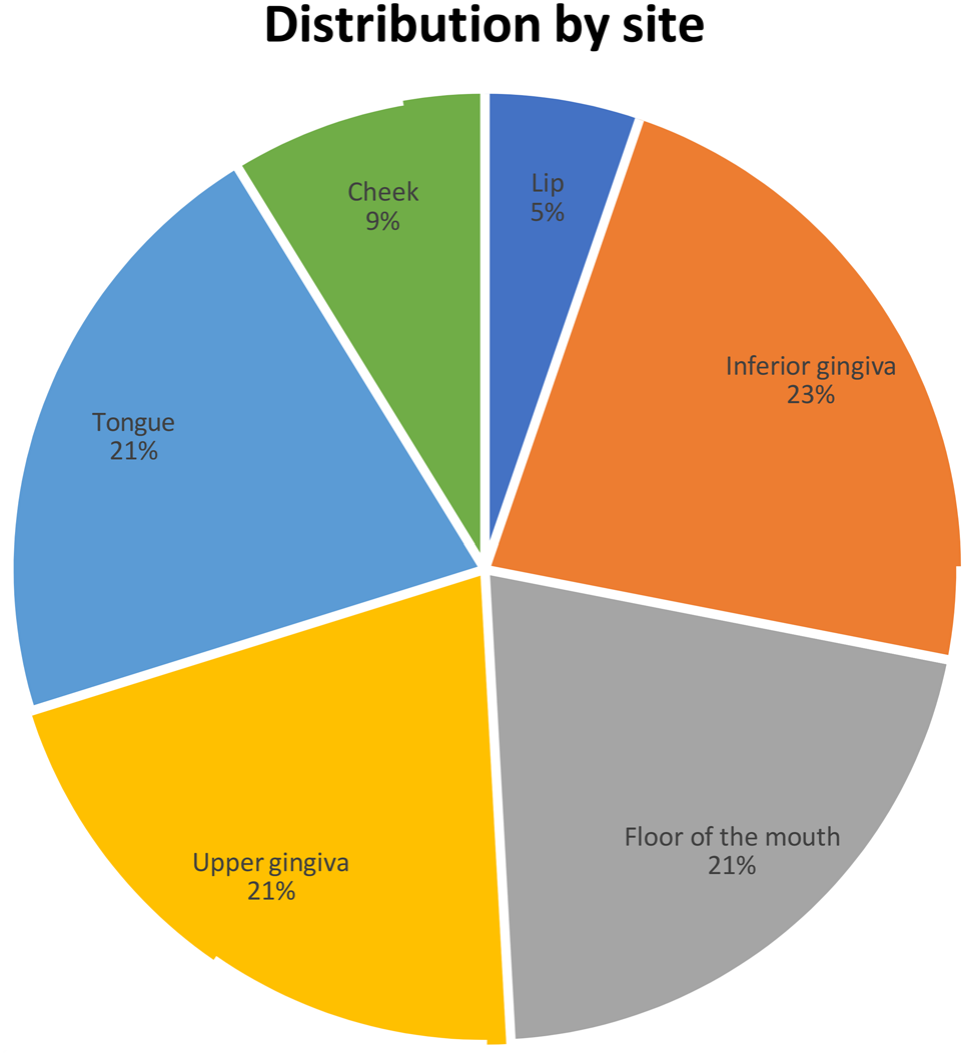
Distribution by site

**Fig. 3:**
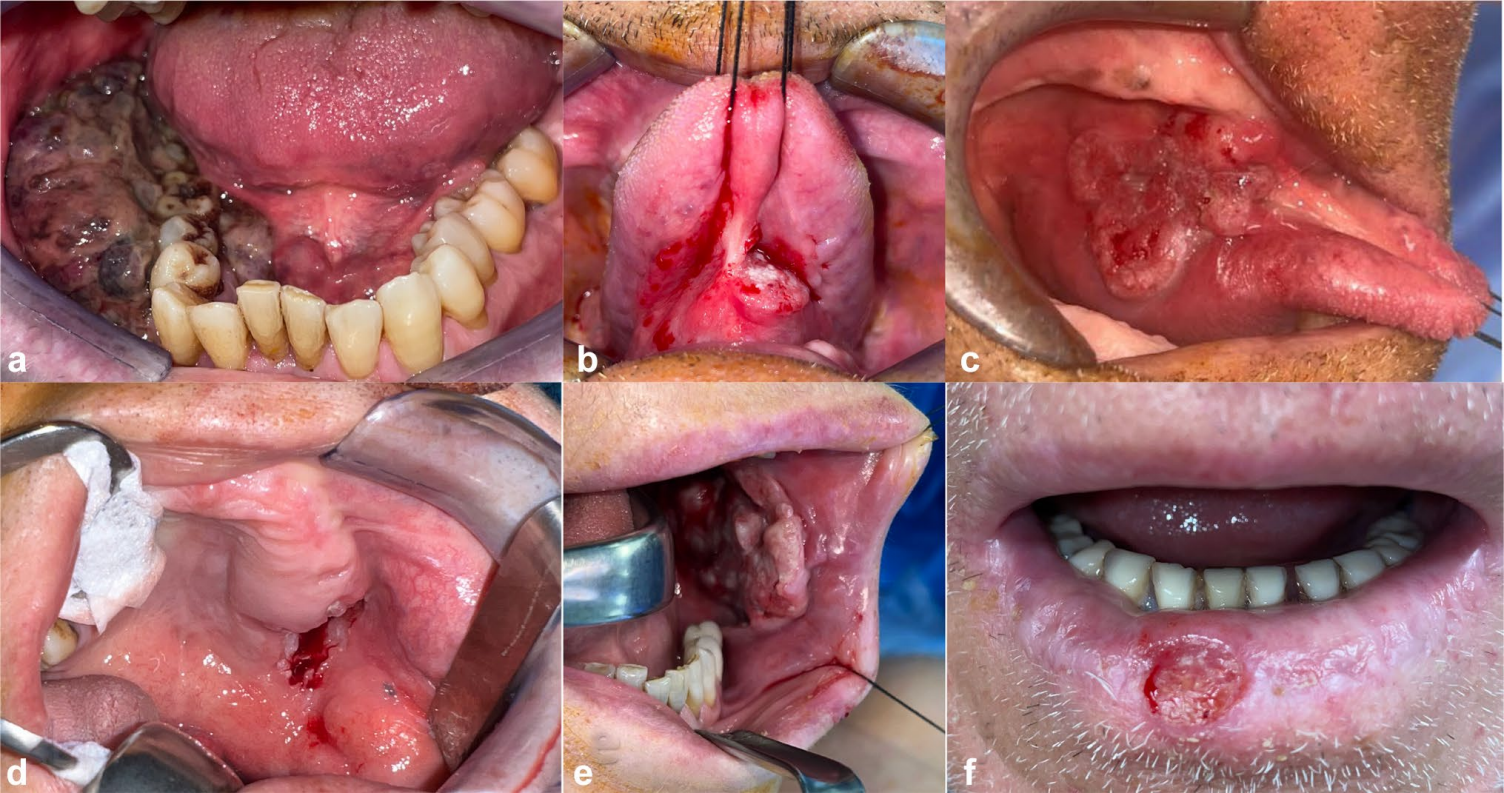
Distribution by the site **a** 23% lower gingiva, **b** 21% floor of the mouth, **c** 21% tongue, **d** 21% upper gingiva, **e** 9% cheek, **f** 5% lip

The distribution by the site of our cases was: 23% lower gingiva, 21% floor of the mouth, 21% tongue, 21% upper gingiva, 9% cheek, and 5% lip (Figs. 2, 3).

During surgery, 70 END were performed, and 210 nodes were analyzed with frozen sections. Among the 70 END, 52 were negative after frozen sections (156 negative nodes), and surgery was ended.

After frozen section examination, the remaining 18 END had at least one positive node. The neck dissection was completed with levels IV and V. Three cases were unclear, and we decided to meet surgery with the dissection of grades IV and V.

The positive nodes were located as follows: 13 level I nodes, 7 level II nodes and 4 level III nodes (24 positive nodes out of 54 examined on 18 END pN+).

All frozen sections were compared with a definitive examination after paraffin inclusion. Five of the 52 opposing ENDs resulted in pN+ after paraffin inclusion (9.6%), which underwent postoperative adjuvant treatment. All 5 cases had only one positive node without any sign of extracapsular spread, 3 in level I, 2 in level II and no nodes were found positive in level III definitive examination. No evidence of level IV and V nodal dissemination was found during the follow-up in these five patients.

All 18 positive frozen sections were confirmed after paraffin inclusion.

**Fig. 4:**
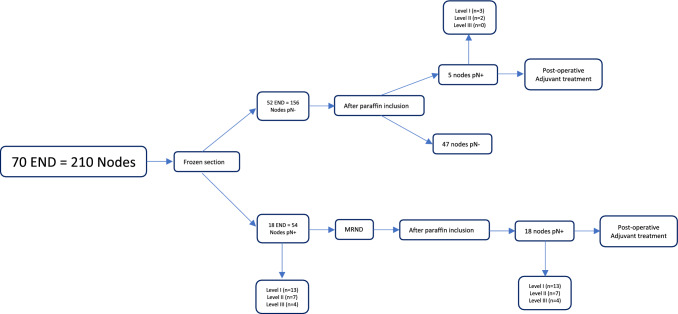
Flow chart after frozen section of the lymph nodes

The 18 frozen section-positive patients underwent completion of levels IV and V. 3 patients (20%) were found metastases in groups IV and V (2 metastases in level IV and one metastasis in level V) (Fig. 4).

The sensibility of our END + frozen section method was 75%, while the specificity of our test was 94%. The negative predictive value was 90,4%.

## Discussion

According to the actual NCCN Guidelines (Rose et al. 2011), the management of the neck in cT1-2N0 patients could follow three different strategies: watchful waiting, elective neck dissection (END) or sentinel lymph node biopsy (SLNB).

The proper neck management of cN0 oral cancer patients has long been controversial.

However, watchful waiting was progressively abandoned until the publication of the papers by D’Cruz et al. (D’Cruz et al. 2015; Bree et al. 2021) in 2015, demonstrating that the relapse rate was higher in cT1-2N0 patients that did not undergo END and the OS and DFS were higher in the END group. The randomized trial compared elective neck dissection (END) versus therapeutic neck dissection to treat cN0 oral cancer. The patients who underwent END were reported to have an improved 3-year overall survival (OS) rate over therapeutic neck dissection (80% versus 67.5%), with an occult metastasis rate of 26.5%.

SLNB provides an intermediate approach between these two options, allowing for the selection of genuinely positive patients for further treatment. On the other hand, in the last decades, the SLNB has spread through the neck management of cT1-2N0 patients after its value was confirmed in treating other tumours, such as melanoma and breast cancer (Bree et al. 2021).

We tried to find an alternative to the SLNB in our department, since, for these methods, a trained and experienced team is needed, and special facilities as the Nuclear Medicine Department is not available in our Hospital. For this reason, we assess the value of the frozen section to spot intraoperatively occult nodal metastases that could have changed the surgical program if detected.

Moreover, SLNB has a diagnostic role for occult metastasis. Still, it is not therapeutic itself, needing a second delayed procedure to perform a therapeutic neck dissection in case of the positive node.

Although SLNB is a reliable technique, it is good to realize that it also has some limitations: SLNB is an invasive technique, and since no reliable intraoperative method to examine the SLNB is yet available, an eventual subsequent neck dissection has to be performed in a second-stage procedure.

Moreover, even if more arbitrary than SLNB, the frozen section technique could overcome the detection of skip metastases that can occur, especially in tongue cancers, and that can also skip the detection of SLNB being performed on the first drainage station. The metastatic nodes involved in OSCC patients are not always the first station drainage nodes. (Bree et al. 2021).

Our END + frozen section method had either a diagnostic role of nodal occult metastases or a therapeutic role. In fact, we already performed an END in the case of a negative frozen section and pN+ at the definitive examination. In contrast, in the case of intraoperative positivity, we completed level IV and V dissection in the same surgical session.

The main disadvantage of an END is the overtreatment of 70 to 80% of the patients with a more invasive procedure. Several studies reported the differences in complication rates, postoperative morbidity and cost-effectiveness in favour of the SLNB compared to the END procedures (Bree et al. 2019)

According to Ross et al. (Ross et al. 2002), SLNB has a sensibility of 90%, whereas our END + frozen section method had a sensibility of 75%.

In general, SLNB has proven to reliably stage the clinically negative neck in early stage OSCC with high sensitivity and negative predictive value (D’Cruz et al. 2015).

However, the sensibility of SLNB lowers to 63% for the floor-of-mouth OSCC [115,20], while our method is not influenced by the primary site (Bree et al. 2021; Toom et al. 2020).

The results of our study showed a specificity of 94%, similar to the values of specificity of the SLNB reported in the literature (93%) (Bree et al. 2021; Toom et al. 2020).

The advantage of our test is that it is a diagnostic and therapeutic one-step surgical procedure but more invasive compared to SLNB, which is also associated with shorter hospitalization (Hernando et al. 2014; Bree et al. 2019; Miura et al. 2017; O’Brien et al. 1995).

In our study, only 3 cases were assessed as false positives, since we had a doubt result at the frozen section examination: completing levels IV and V was uneventful.

Regarding the upbeat frozen section patients, the completion of the IV and V levels revealed occult metastases in these levels in 3 out of 15 patients, allowing us to achieve a more appropriate surgical treatment. Moreover, the recognition of otherwise occult metastases at levels IV and V made it possible to direct radiotherapy treatment to specific targets, which otherwise would have been mainly limited to groups I–II–III.

## Conclusions

Elective neck dissection + intraoperative frozen section could be an excellent alternative to SLNB to spot occult nodal metastases in cT1-2N0 OSCC patients, allowing surgeons to perform a one-step diagnostic/therapeutic procedure. Moreover, an intraoperative positivity could change the surgical strategy and complete level IV and V neck dissection to achieve a more appropriate surgical treatment in the same session.

## Data Availability

If requested, the principal author (Dr Salvatore Battaglia) can provide the data statement.
